# Diverse specificity of cellulosome attachment to the bacterial cell surface

**DOI:** 10.1038/srep38292

**Published:** 2016-12-07

**Authors:** Joana L. A. Brás, Benedita A. Pinheiro, Kate Cameron, Fiona Cuskin, Aldino Viegas, Shabir Najmudin, Pedro Bule, Virginia M. R. Pires, Maria João Romão, Edward A. Bayer, Holly L. Spencer, Steven Smith, Harry J. Gilbert, Victor D. Alves, Ana Luísa Carvalho, Carlos M. G. A. Fontes

**Affiliations:** 1 Centro Interdisciplinar de Investigação em Sanidade Animal, Faculdade de Medicina Veterinária, Universidade de Lisboa, 1300-477 Lisboa, Portugal; 2 NZYTech Genes & Enzymes, Campus do Lumiar, Estrada do Paço do Lumiar, Edifício E, r/c, 1649-038 Lisboa, Portugal; 3 UCIBIO-REQUIMTE, Departamento de Química, Faculdade de Ciências e Tecnologia, Universidade Nova de Lisboa, 2829-516 Caparica, Portugal; 4 Institute for Cell and Molecular Biosciences, Newcastle University, The Medical School, Newcastle upon Tyne NE2 4HH, United Kingdom; 5 Institute of Physical Biology, Heinrich Heine University, Universitätsstr. 1, 40225 Düsseldorf, Germany; 6 Department of Biomolecular Sciences, The Weizmann Institute of Science, Rehovot, Israel; 7 Department of Biomedical and Molecular Sciences, Queen’s University, Kingston, ON K7L 3N6, Canada

## Abstract

During the course of evolution, the cellulosome, one of Nature’s most intricate multi-enzyme complexes, has been continuously fine-tuned to efficiently deconstruct recalcitrant carbohydrates. To facilitate the uptake of released sugars, anaerobic bacteria use highly ordered protein-protein interactions to recruit these nanomachines to the cell surface. Dockerin modules located within a non-catalytic macromolecular scaffold, whose primary role is to assemble cellulosomal enzymatic subunits, bind cohesin modules of cell envelope proteins, thereby anchoring the cellulosome onto the bacterial cell. Here we have elucidated the unique molecular mechanisms used by anaerobic bacteria for cellulosome cellular attachment. The structure and biochemical analysis of five cohesin-dockerin complexes revealed that cell surface dockerins contain two cohesin-binding interfaces, which can present different or identical specificities. In contrast to the current static model, we propose that dockerins utilize multivalent modes of cohesin recognition to recruit cellulosomes to the cell surface, a mechanism that maximises substrate access while facilitating complex assembly.

Cellulosomes are highly efficient nanomachines produced by anaerobic microbes to deconstruct plant structural carbohydrates^1^,^2^,^3^. Cellulose and hemicellulose are the most abundant polymers on earth and thus cellulosomes play a key role in carbon turnover. In addition, cellulosomes are central elements for the production of readily available sugars in the gastrointestinal tract of a variety of mammals^4^. It is now well established that a wide array of highly ordered protein-protein interactions involving dockerin and cohesin modules (defined herein as Doc and Coh, respectively) mediate both cellulosome assembly and cellulosome cell-surface attachment^2^. In *Clostridium thermocellum (Ct*), cellulosome assembly occurs through the binding of type-I Cohs within the primary scaffoldin (ScaA) to type-I Docs located in cellulosomal enzymes^5^,^6^. Primary scaffoldins, in addition to containing multiple type-I Cohs, have a divergent type-II Doc that binds to type-II Cohs located in cell-surface anchoring scaffoldins, providing a mechanism to tether the cellulosome to the bacterial cell envelope^2^,^7^. Coh-Doc complexes exhibit one of the strongest protein-protein affinities known in Nature, and it is evident that the precise assembly and cell surface attachment of cellulosomes are orchestrated by the different specificities displayed by type-I and type-II Coh-Doc interactions, respectively^1^,^8^.

The crystal structures of type-I Coh-Doc complexes revealed a remarkable internal 2-fold structural symmetry that was shown to possess significant functional importance^5^,^9^,^10^. Thus, type-I Docs can bind to their cognate Cohs either through the N- or C-terminal α-helix (helices 1 and 3, respectively), revealing the presence of two near-identical Coh-binding interfaces^5^,^9^. The resultant Doc dual-binding mode is believed to confer considerable flexibility to cellulosome assembly. Remarkably, the crystal structure of the *C. thermocellum* primary scaffoldin (ScaA) type-II Doc in complex with the Coh of the anchoring scaffoldin ScaF revealed striking differences with type-I Coh-Doc complexes^6^. The type-II Doc associates with its neighbouring X-module in ScaA, a module absent in type-I Docs of the cellulosome enzyme subunits. It is thought that the X-module provides the required stabilization of the type-II Doc fold in order to maximize Coh affinity^6^. Unlike type-I Docs, in which ligand recognition is dominated by only one of the Doc helices in either binding modes, in type-II Docs both helices contact the Coh surface over their entire length^6^. Given the involvement of both Doc helices in type-II Coh recognition, and the lack of symmetry in the hydrophobic and hydrogen bond contacts at the type-II Coh-Doc interface, it was proposed that, unlike type-I interactions, type-II Docs present a single Coh-binding mode^6^. However, the current “static” model of the attachment of the cellulosome to the cell surface is based on the crystal structure of only one type-II Coh-Doc complex.

The cellulosomes of *C. thermocellum* and *Acetivibrio cellulolyticus* are highly elaborate macromolecular complexes (Fig. 1). In *C. thermocellum*, the cellulosome is organized by primary scaffoldin ScaA and five different anchoring scaffoldins (herein termed ScaB, ScaC, ScaD, ScaE, and ScaF) (Fig. 1). In addition to ScaA, inspection of *C. thermocellum* genome revealed the presence of an ORF (Cthe_1806) encoding a protein comprising 2177 amino acids and containing a putative type-II Doc, which was termed CipB. Transcriptomic and proteomic studies revealed that this protein is upregulated when *C. thermocellum* is grown on recalcitrant carbohydrates such as cellulose^11^,^12^. CipB contains a signal peptide followed by four modules of unknown function (UNKs), including a region containing 19 repeats of a 41-residue motif with three highly conserved replicated cysteine residues. Upstream of the UNKs is an X-module and the C-terminal type-II Doc. *A. cellulolyticus* cellulosome presents a similar degree of complexity although recruitment of the macromolecular complex to the cell surface may involve the activity of an adaptor scaffoldin (ScaB) as well as typical anchoring scaffoldins (ScaD and ScaF) (Fig. 1).

Cell surface attachment of cellulosomal multi-enzyme complexes is a mechanism of considerable biological importance, although it remains poorly understood. Here, the structures of five different Coh-Doc complexes from two bacteria were elucidated to interrogate the functional complexity of cellulosome recruitment to the cell envelope. In contrast to the previously suggested “static” model, the data support the hypothesis that binding of large cellulosomal complexes to the bacterial surface is mediated by a dual-binding mode that, nonetheless, assumes a diversity of mechanisms in different bacteria.

## Results and Discussion

### Novel components of *C. thermocellum* cellulosome

**Figure 1:**
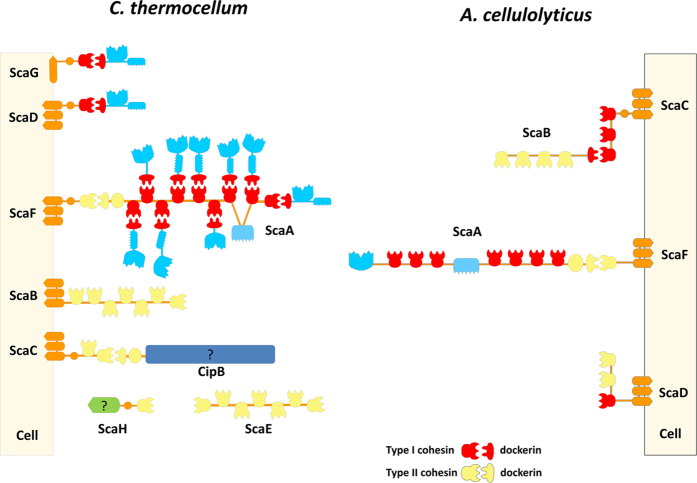
Organization of *C. thermocellum* and *A. cellulolyticus* cellulosomes. *C. thermocellum* scaffoldin CipA, herein renamed ScaA contains nine type I cohesin modules and thus organizes a multi-protein complex comprising 9 enzymes. The C-terminal type II dockerin module of ScaA binds, specifically, type II cohesin modules found in cell surface proteins (ScaB, ScaC and ScaH, previously termed OlpB, Orf2p and SdbA, respectively) or to the extracellular scaffoldins ScaE and ScaH. Since the anchoring scaffoldins ScaB and ScaC and the presumed cell-free scaffoldin ScaE contain more than one type II Coh, they effectively contribute to the assembly of polycellulosomes that may contain up to 63 catalytic subunits in the case of ScaB and ScaE. Nevertheless, cellulosomal enzymes may adhere directly to the bacterial cell surface by binding the single type I Coh found in ScaD and ScaG. Although *A. cellulolyticus* cellulosome is very similar to *C. thermocellum*, it contains an extra adaptor scaffoldin, ScaB, that allows four ScaA scaffoldins to be incorporated in a single cellulosome, thus amplifying the number of enzymes in the complex.

*C. thermocellum* anchoring scaffoldins contain one (ScaF and ScaH), two (ScaC) or seven type-II Cohs (ScaB and ScaE) (Fig. 1). The capacity of ScaA and CipB type-II Docs, fused to their respective X modules (the bi-modular proteins were termed XDocs), to bind to type-II Cohs selected from the five different anchoring scaffoldins, including the two Cohs of ScaC, was investigated. The data revealed that both the ScaA and CipB type-II Docs bound all the type-II Cohs evaluated, although with varying affinities, but were unable to recognize type-I Cohs from ScaA (Figure S1). This demonstrates that the CipB Doc is functional and discriminates type-II from type-I Cohs. The ScaA and CipB XDocs bound equally well and preferentially to ScaC2 Coh, followed by the Cohs of ScaF, ScaH and ScaE6. The six type II Cohs (i.e., ScaB6, ScaC1, ScaC2, ScaE6, ScaF and ScaH) were unable to discriminate between ScaA and CipB XDocs, suggesting that the two type-II Docs bind different type-II Cohs with similar affinities (Figure S1).

### Structure of type-II CipB XDoc in complex with ScaC2 Coh

**Figure 2:**
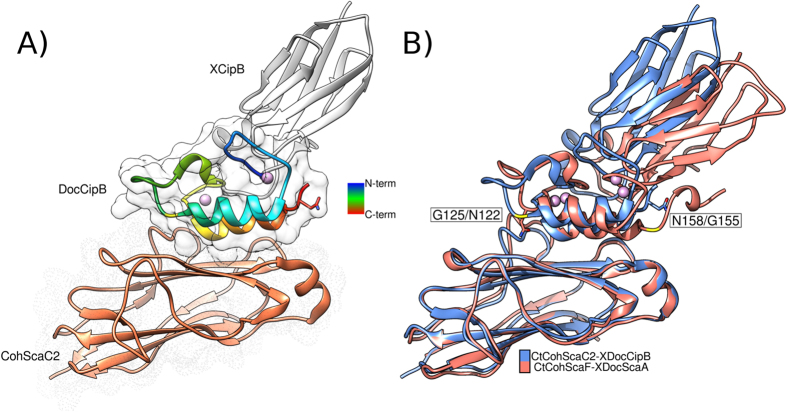
The structure of *C. thermocellum Ct*CohScaC2-XDocCipB complex versus the known structure of *Ct*CohScaF-XDocScaA. The structure of *Ct*CohScaC2-XDocCipB complex was solved (Panel A) and overlaid with the structure of *Ct*CohScaF-XDocScaA (Panel B). In panel A, the Doc structure is rainbow colored, calcium ions are modeled as plum colored spheres and the Coh surface is depicted as dots, whereas the Doc surface is solid white. Relevant residues, Asn158 and Gly125, are shown in stick representation and a contrasting yellow ribbon zone, respectively.

Previously, the structure of ScaF Coh in complex with ScaA XDoc (complex termed *Ct*CohScaF-XDocScaA) was determined^6^. To assess the mechanistic implications for the different Doc affinities to the distinct type-II Cohs, the crystal structure of CipB XDoc in complex with ScaC2 Coh (complex termed *Ct*CohScaC2-XDocCipB–PDB code: 5k39) was solved (Fig. 2). ScaC2 Coh was selected, as both ScaA and CipB Docs were shown to bind preferentially to this Coh (Figure S1). In the complex, the ScaC2 Coh formed a typical flattened, elongated 9-stranded β-barrel with a jelly-roll topology. The crowning α-helix observed between strands 6 and 7 and the two β-flap regions that disrupt the normal progression of strands 4 and 8, respectively, seem to be a common feature of type-II Cohs^13^,^14^. The XDoc dyad was modelled as one single polypeptide chain of 164 amino acids with the first 98 corresponding to the X-module and the remaining to the type-II Doc. The X-module was composed of seven β-strands arranged into two β-sheets (1-4-7 and 2-3-5-6) and a small α-helix connecting strands 1 and 2. The overall fold of this subunit and the β-sheet topology are similar to the X-module of ScaA (PDB code: 2b59), backbone r.m.s.d. of 0.8 Å, and the Ig-like module of avian carboxypeptidase D domain II (PDB code: 1qmu) with a r.m.s.d. of 1.1 Å for the main chain carbons. The CipB type-II Doc (residues 99–164) formed two loop-helix F-hand motifs^15^ separated by a 23-residue linker that also forms a small helix. Both F-hand motif loops coordinated a calcium ion in a typical octahedral geometry. The N- and C-terminal helices (helix 1 and 3, respectively) were arranged in an antiparallel orientation that places the two calcium ions at the opposite ends of the Doc module, similar to that observed for the previously described type-II ScaA XDoc^6^.

The X-module and the type-II Doc form an intimate hydrophobic interface involving a number of Coh and Doc surface residues (Figure S2B). When comparing the XDoc dyad interface of *Ct*CohScaC2-XDocCipB and *Ct*CohScaF-XDocScaA complexes, a more extensive network of contacts was observed between CipB Doc and the X-module. In the *Ct*CohScaC2-XDocCipB complex the X-module stabilized both Doc calcium binding loops, while in the ScaA Coh-XDoc complex the X-module only interacted with the first calcium-binding loop of the Doc (Figure S2A,B). The higher number of contacts at the CipB XDoc interface should lead to a stable and more rigid structure that reduces the entropic cost arising from a tightening of the isolated type-II Doc structure upon type-II Coh binding. Similar to what was observed in the *Ct*CohScaF-XDocScaA complex, both helices of CipB Doc interacted with the planar surface of the ScaC2 Coh comprising β-strands 8-3-6-5 (Fig. 2).

The nature of the ScaC2 Coh:CipB XDoc interacting platform was investigated. A detailed list of contacts observed at the Coh-Doc interface is presented in Table S1. Binding was dominated by Asn146, Leu147 and Phe148 of second Doc repeat. Overall, the interface between the two binding partners in the *Ct*CohScaC2-XDocCipB complex is more intricate than the *Ct*CohScaF-XDocScaA complex^6^. In addition, the CohScaC2-XDocCipB interacting region is predominantly hydrophobic (Table S1). Only two direct hydrogen bonds were identified at the ScaC2 Coh:CipB XDoc interface: Nδ2 of Asn146 Doc and O of Phe136 Coh, and N of Leu147 Doc and Oε1 of Gln37 Coh (Figure S2C,D; Table S1). Furthermore, Asp20 from the X-module formed three water-mediated hydrogen bonds with residues Gln152 and His153 of the Coh (Figure S2E,F). Thus, the data revealed that the interfaces of CohScaF-XDocScaA^6^ and CohScaC2-XDocCipB possess unique physicochemical properties.

Similar to what was observed in the *Ct*CohScaF-XDocScaA complex, the C-terminal helix (helix 3) of CipB Doc dominated Coh recognition. Thus, structures of the *Ct*CohScaA-XDocScaA and *Ct*CohScaC2-XDocCipB protein complexes display a high degree of similarity (Fig. 2B), which is reflected by the low r.m.s.d values when the two complex structures were compared (1.1 Å for 166 Cα atoms of the entire complex, 0.9 Å for 156 Cα atoms of the Coh alone, 0.9 Å for 127 Cα atoms of the XDoc module). To analyze in detail the structural differences between the two type-II complexes, the structures of CohScaF-XDocScaA and CohScaC2-XDocCipB were overlaid (Fig. 2B). Interestingly, Asn122, a key residue present in helix 1 of ScaA Doc that makes three hydrogen bond contacts with the ScaF Coh^6^, is substituted for a glycine (Gly125) in CipB Doc (Fig. 2B). This difference reflects inherent topological differences of ScaF and ScaC2 Cohs. The β-flap interrupting strand 8 of ScaC2 Coh contains Phe136, whose side chain occupies a hydrophobic space positioned towards the centre of the CohScaC2-XDocCipB complex. In ScaF Coh this residue is replaced by a Ser, and the β-flap is placed further away from the Coh-binding interface. This comparison indicates that the side chain of Phe136 in the ScaC2 Coh would clash with the side chain of Asn122 in ScaA Doc, strongly suggesting that the ScaA Doc is unable to bind Coh ScaC2 in this orientation. However, considering the inherent symmetry allowed by the interaction of two Doc helices to the Coh platform, it is possible that, if rotated by 180°, the ScaA Doc would be better positioned to bind ScaC2 Coh, a hypothesis that was further explored below. Conversely, CipB Doc would preferentially bind ScaF Coh through its N-terminal helix as suggested by the primary sequence comparison of the two docs (Figure S1).

### *C. thermocellum* type-II Docs present a single binding mode

The binding thermodynamics of CipB XDoc to its various type-II Coh partners was assessed by isothermal titration calorimetry (ITC). Using ScaC2 or ScaF Cohs as CipB XDoc binding partners, the interaction was found to exceed the limit for accurate determination of the affinity constant, suggesting an association constant (*K*_a_) > 10^9^ M^−1^ (Table 1, Figure S3). The biochemical analysis presented above suggested that the ScaE6 Coh would bind the two *C. thermocellum* type-II Docs with a lower affinity. Thus, ITC experiments were performed using the ScaE6 Coh. The data, presented in Table 1 and exemplified in Figure S3, revealed a *K*_a_ for the ScaE6 Coh-CipB XDoc interaction of ~10^7^ M^−1^ at 318.15 K. It is interesting to note that the apparent hydrophobic nature of the type-II interaction is associated with a gain in enthalpy and a loss in entropy. Since ScaC2/ScaE6 interacting platforms share common properties (see below), it is currently unclear why the thermodynamic forces driving ligand binding are not reflected in the nature of the amino acids that mediate Coh-Doc recognition. The thermodynamic parameters are likely to be influenced by changes in solvation, which cannot easily be explained by static crystal structures.

The importance of key residues in the ScaE6 Coh-CipB XDoc interaction was also probed by ITC. The data (Table 1, Figure S3) revealed that substitution of residues Phe124, Leu147 and Phe148 with alanine in CipB XDoc resulted in the complete abolition of ScaE6 Coh binding. The Phe124Ala and Leu147Ala CipB XDoc variants also displayed no affinity for ScaC2 Coh (Table 1, Figure S3), while Phe148Ala and Ile154Ala exhibited relatively weak affinity for this Coh (*K*_a_ ~ 10^5^–10^6^ M^−1^, which were >10^4^ lower than the wild-type protein; Table 1, Figure S3). In contrast, Met114Ala, Met118Ala and Ser121Ala substitutions in CipB XDoc had little influence on affinity for both ScaE6 and ScaC2 Cohs. In contrast, the affinities of wild-type CipB XDoc and all its mutant variants for ScaF Coh were too high to be quantified. To ensure that changes in amino acid composition have not affected the thermostability of protein mutant derivatives at 45 °C, the capacity of the different dockerins to interact with ScaF, ScaC2 and ScaE6 was also probed by native page after incubating the protein partners at the temperature used in the ITC experiments (Figure S4). Overall, these data suggest that Phe124, Leu147 and Phe148 in the CipB Doc module are essential for ScaC2 and ScaE6 Coh recognition. Thus, in contrast to type-I Docs that present two Coh-binding interfaces displaying similar affinities (hence single Ala substitutions in the Doc has no influence on affinity due to substantial functional redundancy), it is proposed that CipB XDoc only presents a single ScaC2/ScaE6 Coh-binding surface. The fact that CipB XDoc variants that fail to bind ScaC2/ScaE6 retain affinity for the ScaF Coh, suggests that the type-II Doc interacts with different Cohs through distinct mechanisms.

### *C. thermocellum* type-II Docs present two different Coh-binding platforms

**Figure 3:**
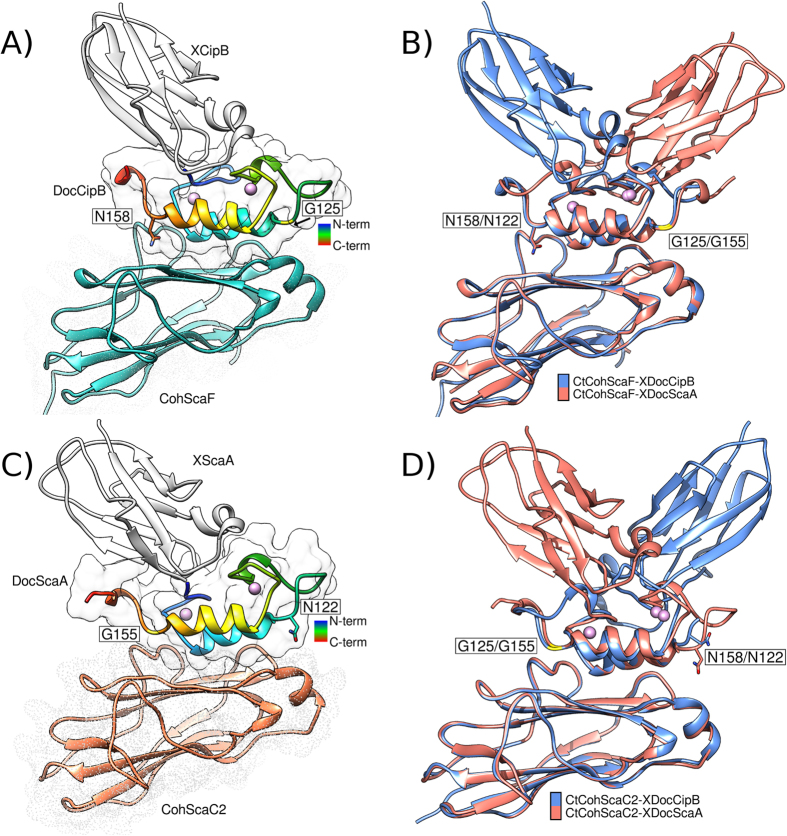
The structures of *C. thermocellum Ct*CohScaF-XDocCipB and *Ct*CohScaC2-XDocScaA complexes. The structures of *Ct*CohScaF-XDocCipB (Panel A) and *Ct*CohScaC2-XDocScaA (Panel C) complexes were solved, illustrated using the same design described in Fig. 2. The structures of *Ct*CohScaF-XDocCipB and *Ct*CohScaF-XDocScaA complexes (Panel B) and of *Ct*CohScaC2-XDocScaA and *Ct*CohScaC2-XDocCipB complexes were overlaid (Panel D).

Data presented above suggest that *C. thermocellum* type-II Docs present two different Coh-binding faces, each of which binds to a different type-II Coh scaffold. Thus, in order to probe the mechanism by which CipB XDoc binds to ScaF and ScaA XDoc recognises ScaC2, the crystal structures of ScaF XDoc in complex with ScaC2 Coh (*Ct*CohScaC2-XDocScaA) and CipB XDoc bound to ScaF (*Ct*CohScaF-XDocCipB) were solved. Strikingly, the data revealed that the two Docs bound ScaF and ScaC2 in different orientations (Fig. 3). Thus, ScaA (helix 3) and CipB (helix 1) XDocs bound ScaF Coh in opposite orientations (Fig. 3A,B). In contrast, ScaC2 Coh recognized ScaA XDoc, predominantly through helix 1 and CipB XDoc through helix 3 (Fig. 3C,D).

Superposition of the two Coh-Doc binding interfaces allowed visualization of the different binding modes displayed by CipB and ScaA XDoc to ScaF or ScaC2, respectively (Fig. 3B,D). When the two complexes comprising ScaC2 Coh were overlaid (*Ct*CohScaC2-XDocScaA and *Ct*CohScaC2-XDocCipB), it was evident that the XDoc modules were in reverse orientations highlighted by the X-modules, which were positioned in opposite directions (Table S1, Fig. 3D). Both ScaC2 Coh and the type-II Docs display a high degree of similarity when these protein modules were independently compared, which is reflected by the low r.m.s.d values (0.5 Å for 160 Cα atoms of the Coh alone and 1.1 Å for 54 Cα atoms of the Doc module). Although the nature of the hydrophobic interactions is almost indistinguishable, the ScaA Doc, which binds ScaC2 predominantly through helix 1, makes an extra hydrogen bond to Phe136 through Ser108 (Table S1). In addition, the binding mode of CipB Doc positions the X-module in closer proximity to the Coh. These differences lead to the formation of three extra water-mediated hydrogen bonds between the X-module and ScaC2 Coh that are absent when the ScaA Doc binds to this cohesin in the opposite orientation (Table S1).

Inspection of the Coh-Doc interface when ScaF is the Coh partner reveals a much more extensive hydrogen-bonding network when compared to the complexes involving ScaC2 Coh (Table S2, Fig. 3B). This observation suggests that the higher affinity displayed by ScaA and CipB XDocs for ScaC2 Coh is a result of the more extensive hydrophobic contacts with this Coh. When *Ct*CohScaF-XDocScaA and *Ct*CohScaF-XDocCipB complexes were overlaid (Fig. 3B) a high structural similarity between the Doc and Coh structures was observed (0.6 Å for 166 Cα atoms of the Coh alone and 1.0 Å for 55 Cα atoms of the Doc module). Doc residues that bind ScaF Coh were highly conserved with the exception of Val115 and Ser121 from CipB XDoc, which correspond to Gln145 and His151, respectively, in ScaA XDoc. ScaA XDoc makes two extra hydrogen bonds with the ScaF Coh through Asn122 and His151, which are compensated in the CipB XDoc-ScaF Coh complex by extra polar contacts established between the side chain of Arg104 of CipBXDoc with Gly166 of ScaF Coh. In addition, as observed for the CipB XDoc bound to ScaC2, when the ScaA XDoc contacted ScaC2 Coh through helix 3, Ser20 of the X-module also made a hydrogen bond with Glu167 of ScaF Coh (Table S2). Thus, binding of the Docs through helix 3 allows the X-module to be in closer proximity to the Coh and to establish polar contacts with this module. Another significant difference in the interaction with ScaF or ScaC2 Coh platforms is the role played by Doc residues Asn158/Asn122 and Gly125/Gly155 in modulating Coh specificity. Binding to ScaF Coh involves the establishment of very important polar contacts between the side chains of Asn158/Asn122 with the Coh (Fig. 3C). Steric constraints do not accommodate Asn158/Asn122 in binding ScaC2 and thus these residues are replaced by Gly125/Gly155 when binding to this Coh (Fig. 3D).

**Figure 4:**
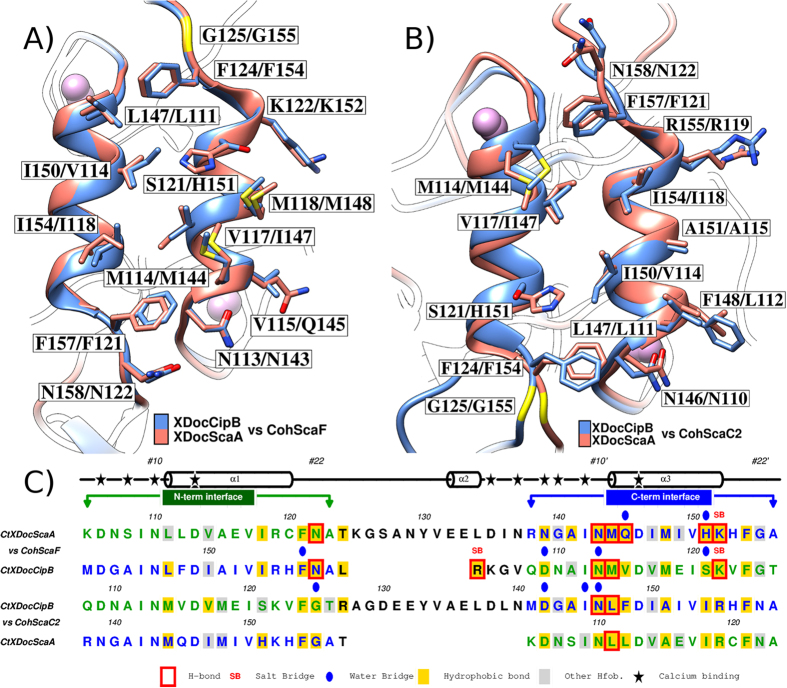
*C. thermocellum* dockerin interfaces that recognize ScaF (Panel A) and ScaC2 (Panel B) Cohs. The important cohesin contact residues are identified and displayed in stick representation. The primary sequence alignment of CipB and ScaA dockerins is provided, highlighting the residues involved in polar (direct and water-bridged), hydrophobic and calcium binding contacts. Residues included in the N-terminal Coh binding interface are shown in green while those integrated in the C-terminal interface are blue (Panel C).

To summarize, the structural and biochemical data here show that the ScaA and CipB XDoc modules bind to ScaC2 and ScaF Cohs in opposite orientations, and different helices thus dominate recognition of the different Cohs. It is evident that the two XDocs contain two distinct Coh-binding sites with distinct specificities. In Fig. 4 the different nature of the two distinct Coh-binding platforms identified in *C. thermocellum* ScaA and CipB Docs is represented. The binding site for ScaF is located primarily in helix 3 of ScaA XDoc and helix 1 of CipB XDoc (Fig. 4A) and is more polar (Fig. 4C). The opposite helix in the two type-II Docs dominates binding to ScaC2 (Fig. 4B). This interaction is predominantly hydrophobic (Fig. 4C). The different binding modes displayed by the two Docs are consistent with striking differences in the primary sequence of the Coh-binding regions of ScaA and CipB Docs (Fig. 4C). For example, positions 11 and 12 of the second calcium-binding loop of ScaA Doc, which were previously shown to display a key role in the recognition of the ScaF Coh in the *Ct*CohScaF-XDocScaA complex, present different physicochemical properties^6^; the ScaA Met144/Gln145 duplet is replaced by a Leu147/Phe148 pair in the CipB Doc. However, if ScaA Doc is aligned with CipB Doc with the two duplicated segments inverted, then a higher degree of conservation between the Coh-interactive residues is apparent (Fig. 4C). These alignments are consistent with the observation that the opposite helix in the ScaA and CipB Doc fulfil an equivalent role in Coh recognition (Fig. 4C). The alignment presented in Fig. 4C emphasizes the important contacts observed when Docs bind to ScaF or ScaC2 Coh platforms.

### *Acetivibrio cellulolyticus* type-II Docs involved in cell surface attachment present a dual-binding mode

The two distinct Coh-binding sites of type-II Docs that assemble cellulosomes on the bacterial surface of *C. thermocellum* may allow plasticity in Coh recognition. In contrast to the sequence and structural asymmetry of *C. thermocellum* type-II Docs, *A. cellulolyticus* type-II Doc, located in its primary scaffoldin ScaA, displays nearly identical segment repeats. These are reminiscent of the highly symmetrical Coh-binding sites in type-I Docs from *C. thermocellum* and other clostridia. It is possible that recruitment of multi-enzyme complexes to the cell surface of *A. cellulolyticus* occurs through a different mechanism when compared to *C. thermocellum*. To probe this hypothesis the structure of *A. cellulolyticus* type-II XDoc in complex with a ScaB Coh was solved. Although ScaB is an adaptor scaffoldin, ScaB Cohs are highly similar to those present in cell surface anchoring scaffoldins ScaD and ScaF and, thus, this type-II complex exemplifies the interactions that recruit the cellulosome to the cell surface of *A. cellulolyticus* (Fig. 1).

**Figure 5:**
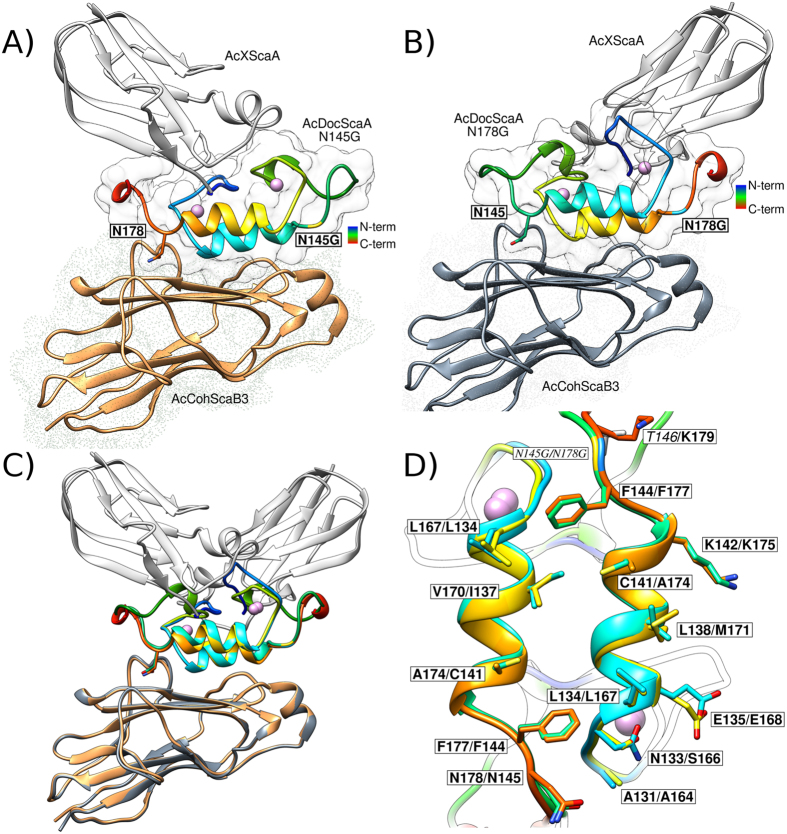
The structure of *A. cellulolyticus* type II Coh-Doc complexes. The structures of *Ac*CohScaB3_XDocScaAN145G and *Ac*CohScaB3_XDocScaAN178G complexes are displayed in Panels A and B, respectively, using the same design described in Fig. 2. The structures of the two complexes were overlayed (Panel C) and the dockerin platform that interacts with ScaB3 Coh was visualized (Panel D).

Initial attempts to crystallize an *A. cellulolyticus* type-II Coh-Doc complex comprising the ScaA XDoc dyad and a Coh from the ScaB subunit failed. This may be due to a dual Doc binding plasticity that would limit the required protein-complex homogeneity needed for protein crystallization. In order to promote a single-binding mode between the two protein partners and enable complex crystallization, two variants of the *A. cellulolyticus* type-II Doc were produced in which the Asn residues at positions 145 and 178 were independently substituted to a Gly (*Ac*XDocScaAN145G and *Ac*XDocScaAN178G; Table S3). The rationale of this substitution was derived from the observations that in *C. thermocellum* a similar alteration modulated Coh specificity (see above discussion). Diffracting crystals of the ScaB3 Coh in complex with either ScaA XDoc variants were obtained. The structures of the *A. cellulolyticus Ac*CohScaB3_XDocScaAN145G (PDB code: 4u3s) and *Ac*CohScaB3_XDocScaAN178G (PDB code: 4wi0) complexes were solved by molecular replacement to a resolution of 1.64 Å and 1.93 Å, respectively (Fig. 5A,B). Analysis of these two complexes revealed that the X-module appended to the type-II Doc was positioned in two different orientations suggesting that the Doc is rotated by 180° in the two structures (Fig. 5C). Furthermore, the engineered substitutions at positions 145 or 178 of the ScaA XDoc did not affect the integrity of the Coh-Doc interacting surface, since the non-mutated Asn residue (at position 145 or 178) interacts with the ScaB3 Coh and the engineered glycine is located at a non-interacting region.

The *Ac*CohScaB3 structure in complex with either XDocScaAN145G or XDocScaAN178G is very similar to the structures of unbound *A. cellulolyticus* type-II Cohs^14^,^16^,^17^,^18^, suggesting that type-II Cohs do not undergo major structural changes upon binding. Similar to the *C. thermocellum* ScaF and ScaC2 type-II Cohs^6^, *Ac*CohScaB3 displays an elongated jelly-roll topology in a nine-stranded flattened β-sandwich structure, defined by two β-sheets, disrupted by additional secondary structures: a unique crowning α-helix between β-strands 6 and 7 and two β-flap regions that interrupt β-strands 4 and 8 (Fig. 5). The X-module located in the proximity of *A. cellulolyticus* type-II Doc is structurally very similar to the *C. thermocellum* homologues. A large hydrophobic interface between the X-module and *A. cellulolyticus* type-II Doc involves twelve X-module residues, while six were involved in polar interactions (Table S4). In the *A. cellulolyticus* ScaA type-II Doc, the highly conserved Doc Ca^2+^-binding site possesses some unique features on the first EF-hand Ca^2+^-binding loop. These differences are a consequence of an unusual four residue insertion (Ile124, Gly125, Gly126 and Thr127) that forms a type IV beta-turn motif, and also the presence of two bridged structural water molecules, instead of the usual single-bridged water. The Doc residues involved in Coh recognition are located over the entire lengths of helices 1 and 3 (Table S4), similar to the type-II Docs of *C. thermocellum*^6^. When considering the angle between the plane defined by the Coh-binding plateau and the main longitudinal axis of the Doc helices, it is more than 30° in the canonical *C. thermocellum* type-I Coh-Doc, whereas in type-II *A. cellulolyticus* Coh-Doc complexes presented here it ranges from 6 to 15°, supporting an almost parallel arrangement in the latter.

An overlay of the two *A. cellulolyticus* complexes (Fig. 5C,D) revealed high conservation of the critical residues located at the two Coh-Doc interfaces and suggested a dual-binding mode in *A. cellulolyticus* type-II Coh-XDoc complexes. While the Coh-Doc interfaces comprise a network of hydrogen bonds and salt-bridge interactions, they also display significant hydrophobic properties (Table S4). Doc residues located at positions 11 and 12 of helix 1 and helix 3 (i.e., Leu134/Leu167, Glu135/Glu168) displayed a central role at the interface, where they contributed to both its hydrophobic and polar character. The complete list of hydrophobic and polar contacts in the two complexes is summarized in Table S4. In *A. cellulolyticus* (in both orientations) and *C. thermocellum* Coh-Doc complexes involving ScaF XDoc, there is a notable conservation among the main interactions of the XDoc with their cognate Coh partners. Overall, these observations provide a structural rationale for the promiscuous nature of type-II interactions between *C. thermocellum* and *A. cellulolyticus*. A noteworthy difference in the *A. cellulolyticus* Coh-Doc complex is the absence of direct hydrogen bonds between the X-module and the Doc module, as observed in *C. thermocellum* type-II complexes^6^.

## Conclusions

This study reveals that cellulolytic anaerobic bacteria, notably *C. thermocellum* and *A. cellulolyticus*, employ different molecular mechanisms to attach elaborate cellulosomal complexes to their bacterial cell surfaces. Type-II Docs of primary scaffoldins ScaA and CipB in *C. thermocellum* possess two distinct Coh-binding surfaces that recognize different Coh partners. These diverse specificities allow a single but distinct Doc binding mode to the different Cohs presented on the surface of this bacterium. In contrast, suppleness in Coh recognition is a dominant feature of the type-II Doc module of *A. cellulolyticus*, where a dual-binding mode was observed. The latter mechanism of Coh-Doc recognition would impose additional structural freedom to ensure efficient assembly of these elaborate complexes and the corresponding degradation of plant cell walls.

## Methods

### Cloning, expression and purification of unbound proteins and protein complexes

Docs are characteristically unstable when expressed in *Escherichia coli*^8^. Therefore, all complexes were obtained by co-expressing in *E. coli* the genes encoding Docs with their Coh counterparts. Co-expression of Docs and Cohs allows the immediate binding of Docs with their interacting partners leading to protein stabilization (2). Genes encoding Cohs and Docs were cloned into pET28a (Novagen) under the control of separated T7 promoters and terminators. Genes were designed with a codon usage optimized for expression in *E. coli* and synthesized (NZYTech, Portugal). Synthetic nucleic acids were synthesized comprising the following DNA sequences (from the 5′ to the 3′end): Doc, T7 terminator, T7 promoter, Coh. The artificial nucleic acids contained two engineered restriction sites at each 5′and 3′-ends, respectively, allowing cloning into pET28a in two different forms. In one form the engineered hexahistidine tag was located at the N-terminus of the Doc (cloning using Nhe I–Sal I sites); in the second the affinity tag was positioned at the C-terminus of the Coh (cloning using NcoI–XhoI sites). Differential cloning into pET28a resulted in Coh-Doc complexes that contained either the hexahistidine tag in the Doc or the Coh. Thus, for the six Coh-Doc complexes produced in this study, two pET28a derivatives were obtained leading to a total of twelve recombinant plasmids generated. The plasmids were used to transform *E. coli* BL21 (DE3) cells using standard protocols. Levels of expression of the twelve Coh-Doc derivatives were determined by purifying complexes through immobilized metal ion affinity chromatography (IMAC) as described below. For *C. thermocellum* Coh-Doc complexes were expressed at higher levels when the engineered tag was located at the Coh. In contrast, *A. cellulolyticus* protein complexes were better expressed when the hexahistidine tag was engineered in the Doc. The primary sequences of recombinant Coh-Doc complexes generated in this study are available in Table S3. In addition, Cohs and Docs used for protein binding studies were also expressed individually in *E. coli*. Genes encoding Cohs were cloned in pET28a (NheI–XhoI) and the recombinant protein contained an engineered N-terminal hexahistidine tag. In contrast, nucleic acids encoding bacterial Docs and their neighboring X-modules were cloned in pET21a (NdeI–XhoI) and the corresponding protein contained an engineered C-terminal hexahistidine tag. The primary sequences of all Cohs and Docs analysed in this study are available in Table S5. *E. coli* Tuner (DE3) cells, transformed with pET21a derivatives, and BL21 (DE3) cells, transformed with pET28a derivatives, were grown at 37 °C to mid-exponential phase (OD_600_ = 0.5). Recombinant protein expression was induced by adding 1 mM IPTG (isopropyl β-D-thiogalactoside) and cells were further cultivated at 19 °C for 16 h. Soluble recombinant proteins and protein complexes were purified by IMAC as described previously^5^. Individual Cohs and Docs used for protein binding studies were buffer exchanged, using PD-10 Sephadex G-25M gel filtration columns (GE healthcare), into 50 mM HEPES pH 7.5, 2 mM CaCl_2_ and 0.5 mM TCEP. For crystallography, protein complexes were further purified by anion exchange chromatography using a Source 30Q column and a gradient elution of 0–1 M NaCl. This allowed separating the complex from unbound Cohs or Docs. Fractions containing the protein complex were buffer exchanged and then concentrated in 0.5 mM CaCl_2_ to 15 to 30 g/L.

### Site-Directed Mutagenesis

Site-directed mutagenesis was carried out employing a PCR-based NZYMutagenesis kit (NZYTech, Portugal) using the plasmids encoding the appropriate Cohs or Docs as template. Primers used to generate the mutant DNA sequences are displayed in Table S6. The mutated DNA clones were sequenced to ensure that only the appropriate DNA change was accumulated after the PCR.

### Non-denaturing PAGE

Complex formation was initially evaluated through non-denaturing PAGE^9^. The two putatively interacting proteins were combined in 50 mM HEPES pH 7.5, 100 mM NaCl and 5 mM CaCl_2_, for 1 h at 25 or 45 °C, and complex formation was further analysed in 10% (v/v) non-denaturing polyacrylamide gels. For lower-affinity interactions, complexes were detected by increasing concentrations of a Doc against a fixed concentration of Coh. New bands appearing in the native gel were used as an indication of complex formation.

### Isothermal Titration Calorimetry (ITC)

The thermodynamics of the various protein-protein interactions were quantified by ITC using a VP-ITC calorimeter (MicroCal, Northampton, MA, USA), as described previously^9^. Titrations were carried out 318.15 K. During titration, the Doc was stirred at 300 rev/min in the reaction cell, which was injected with 28 successive 10 μL aliquots of ligand comprising Coh at 300 s intervals. Integrated heat effects, after correction for heats of dilution, were analysed by non-linear regression using a single site-binding model (Microcal ORIGIN, Version 7.0; MicrocalSoftware). The stoichiometry of binding (n), the association constant (*K*_a_) and the binding enthalpy ΔH were evaluated by using MicroCal Origin software. The standard Gibbs energy change ΔG_0_ and the standard entropy change ΔS_0_ were calculated using the thermodynamic equation − RTlnK_A_ = ΔG = ΔH − TΔS where R is the gas constant and T the absolute temperature.

### Complex crystallization and X-ray data collection and processing

#### Clostridium thermocellum complexes

Protein complexes were crystallized at 293 K using the sitting-drop vapour-phase diffusion method with an equal volume (1 μL) of protein and reservoir solution (unless otherwise stated), using a robotic nanodrop dispensing systems (Oryx8, Douglas Instruments). Crystals of the *Ct*CohScaC2-CipBXDoc were grown in 4% Tacsimate, pH 5.0, and 12% PEG 3350 over a period of 3–5 days and were cryoprotected with 30% glycerol. The space group was determined to be *C*121 with unit cell dimensions *a* = 116.67 Å, *b* = 78.63 Å, *c* = 35.80 Å, with β = 95.87°, which corresponds to a Matthews coefficient of 2.2 Å^3^ Da^−1^ for one *Ct*CohScaC2-CipBXDoc heterodimer in the asymmetric unit, with a solvent content of 43.13%. Crystals of the *Ct*CohScaF-XDocCipB were grown in 30% PEG 400, 0.2 M (NH_4_)_2_SO_4_ and 0.1 M acetate pH 4.5 and were cryoprotected with 25% glycerol. Crystals grew in space group *P*2_1_2_1_2_1_ with unit cell dimensions *a* = 43.41 Å, *b* = 63.74 Å and *c* = 141.20 Å, which corresponded to a Matthews coefficient of 2.64 Å^3^ Da^−1^ for one *Ct*CohScaF-XDocCipB heterodimer in the asymmetric unit and solvent content of 53%. Crystals of the *Ct*CohScaC2-XDocScaA were grown in 25% PEG 3350, 0.2 M CaCl_2_ and 0.1 M HEPES pH 7.5 and were cryoprotected with 30% glycerol. Crystals grew in space group *I*222 with unit cell dimensions *a* = 52.36 Å, *b* = 125.80 Å and *c* = 130.47 Å, which corresponded to a Matthews coefficient of 3.1 Å^3^ Da^−1^ for one *Ct*CohScaC2-XDocScaA heterodimer in the asymmetric unit and a solvent content of 60%. The data of the three complexes were obtained from single crystals at the European Synchrotron Radiation Facility (ESRF) in Grenoble, France. For *Ct*CohScaC2-XDocCipB, data collection was performed at a wavelength of 0.9735 Å in ID14-EH4, while for *Ct*CohScaF-XDocCipB and *Ct*CohScaC2-XDocScaA the wavelengths were 0.9762 Å and 0.9537 Å, respectively, in ID29 at a temperature of 100 K. The crystals diffracted to 1.98 Å (*Ct*CohScaC2-XDocCipB), 1.5 Å (*Ct*CohScaF-XDocCipB) and 3 Å (*Ct*CohScaC2-XDocScaA) resolution. Data from *Ct*CohScaC2-XDocCipB were processed and scaled with the software MOSFLM^19^ and SCALA from the CCP4 suite^20^. Data collection and refinement statistics for the three *C. thermocellum* Coh-Doc complexes here reported are summarized in Table S7. In addition, both data from *Ct*CohScaF-XDocCipB and *Ct*CohScaC2-XDocScaA were processed and scaled with the software XDS^21^ and AIMLESS^22^.

#### Acetivibrio cellulolyticus complexes

Various crystallization conditions were screened for *A. cellulolyticus* Coh-Doc complexes using the sitting-drop vapour-phase diffusion method on the same Oryx8 robotic nanodrop dispensing system. Initial essays failed to generate any crystals until two variant forms designed to promote a single binding mode of the Coh-Doc complex were used. Crystals were obtained at 293 K in drops of 0.7 μl of *Ac*CohScaB3-XDocScaAN145G concentrated to 10 or 20 mg/ml and 0.7 μl of reservoir solution with 0.1 M CHES pH 9.5, 20% v/v PEG 8000 and in drops of 1 μl of *Ac*CohScaB3-XDocScaAN178G concentrated to 15 or 26 mg/ml and 1 μl of reservoir solution with 25% PEG 3350, 0.1 M Bis-Tris pH 5.5, 0.2 M MgCl_2_. Crystals were cryo-cooled in liquid nitrogen after being cryoprotected with paratone or 30% glycerol. Data collection for *Ac*CohScaB3-XDocScaAN145G took place at beamline IO3 at Diamond Light Source synchrotron (DLS, Harwell, UK) at 100 K using a Dectris Pilatus 6M-F detector at a wavelength of 0.976 Å. The data set was integrated and scaled with XDS^21^ and AIMLESS^22^ from the CCP4 suite^20^. The crystals belonged to the *P*6_5_22 space group, with cell constants *a* = *b* = 72.32 Å and *c* = 231.49, α = β = 90°, γ = 120°, which corresponded to a Matthews coefficient of 2.31 Å^3^ Da^−1^ for one *Ac*CohScaB3-XDocScaAN145G heterodimer in the asymmetric unit and solvent content of 46.9%, and diffracted beyond 1.64 Å resolution. Data collection for *Ac*CohScaB3-XDocScaAN178G took place at beamline ID29 at the European Synchrotron Radiation Facility (ESRF, Grenoble, France) at 100 K using a Dectris Pilatus 6M-F detector at a wavelength of 0.976 Å. The data set was integrated and scaled with MOSFLM^19^ and AIMLESS^22^ from the CCP4^20^ suite. The crystals belonged to the *P*2_1_2_1_2_1_ space group, with cell constants *a* = 38.54 Å, *b* = 88.30 Å and *c* = 92.02 Å, α = β = γ = 90°, which corresponded to a Matthews coefficient of 2.25 Å^3^ Da^−1^ for one *Ac*CohScaB3-XDocScaAN178G heterodimer in the asymmetric unit and solvent content of 45.3%, and diffracted beyond 1.93 Å resolution (data collection and refinement statistics for the two *A. cellulolyticus* Coh-Doc complexes here reported are summarized in Table S8).

### Structure determination, refinement and model building

#### Clostridium thermocellum complexes

The crystal structure of the *Ct*CohScaC2-XDocCipB complex was solved by molecular replacement using the program PHASER from the CCP4 suite^20^ and the ScaF type-II Coh-XDoc complex (PDB ID code: 2b59) as model. Data, up to 1.98 Å resolution, were used for structure refinement and the final statistics are summarized in Table S7. Initial building of the complex into the electron density was performed using ARPwARP^23^, and the remaining residues were manually built using COOT^24^. The refinement was performed using REFMAC5^23^. Water molecules were added and final refinement included translation, liberation and screw-rotation of the two independent groups (molecules A and B). The final model has R_work_ = 18.7% and R_free_ = 24.7% and includes 322 water molecules and two calcium ions. The residues Met1 and Ala1 of the Coh module (chain A), Met1, Asn2, Asn3, Asp4, Ser5 and Thr6 of the X-module (chain B) and Leu160, Pro161, Ser162, Arg163 and Tyr164 from the Doc module (chain B) were not observed. The side chains of residues Arg73, Lys158 and terminal hexahistidine tag from the Coh module and Glu63 and Lys85 from the X-module were also not observed. The structure was deposited in the Protein Data Bank under the accession code: 5k39. All polypeptide chains are well defined in the electron density map (with the exception of the residues mentioned above) with average B factors of 16.6, 16.7 and 16.6 Å^2^ for the Coh, X- and Doc modules, respectively. The structure of *Ct*CohScaF-XDocCipB complex was solved by molecular replacement using the program PhaserMR^25^ implemented in the PHENIX^26^ suite using as models the ScaF type-II Coh (PDB ID code: 2bm3) and the Doc from the complex *Ct*CohScaF-XDocScaA (PDB ID code: 2b59). The solution containing the Coh and X-module was inserted in PHENIX Autobuild^26^ for automated building of the CipB Doc. Further model building refinement was performed using COOT^24^, REFMAC5^23^ and PDB_REDO. The final model contains in addition to the two macromolecular chains, two calcium ions and 322 water molecules. In chain A, five residues from the N-terminus and six residues from the C-terminus were not observed in the electron density and in chain B the first six residues from the N-terminus. The structure of the complex was deposited in Protein Data Bank with the accession code 5m0y. Phasing of the *Ct*CohScaC2-XDocScaA complex from *C. thermocellum* was solved by molecular replacement using the program PhaserMR^25^ and the ScaF type-II Coh from *C. thermocellum* (PDB ID code: 2bm3) as model. A clear solution was found with an LLG = 1362.61 and TFZ = 23.6. PHENIX Autobuild^26^ was used for model improvement and completion to 3.0 Å resolution, building the XDocScaA given the primary sequence. The R_work_ and R_free_ values after Autobuild were 24.8% and 33.9%, respectively. Iterative model building and refinement were performed using COOT^24^, REFMAC5^23^ and finally PDB_REDO to final values of R_work_ = 25.6% and R_free_ = 30.3%. Besides the CohScaC2 and XDocScaA modules, the final model includes two calcium ions. For the CohScaC2 module, the N-terminal Met-Ala-Ser-Ala region and the Lys-Ile-Thr-Val-Ile C-terminus, were not observed in the electron density and were thus omitted from the final model. As for the XDocScaA Doc module, the 19-residue tag plus six residues from the N-terminus were not built in the electron density, as well as the final C-terminus Gln residue. The corresponding set of coordinates is deposited in the Protein Data Bank under the accession code 5g5d. Data collection and refinement details data for the three complete structures are summarised in Table S7.

#### Acetivibrio cellulolyticus complexes

The crystal structure of the *Ac*CohScaB3-XDocScaAN145G complex was solved by molecular replacement using the program BALBES^27^ from the CCP4 suite^20^ using as models the structures of PDB ID codes 2b59 from *C. thermocellum* (5) and 3fnk and 3l8q from *A. cellulolyticus* (6, 7). Data up to 1.64 Å resolution were used for structure refinement and the final statistics are summarized in Table S8. Initial building of the complex into the electron density was performed using ARPwARP^23^ and the remaining residues were manually built using COOT^24^. Refinement was done with REFMAC5^23^ and PDB_REDO. Water molecules were added/validated according to the following criteria: a compatible water-shaped peak (F_o_ − F_c_ > ~3 σ, 2F_o_ − F_c_ > ~1.5 σ), whose center was within acceptable hydrogen bond distance to the closest protein atoms or other waters (~2.4–3.2 Å), and B-factors similar to neighboring atoms but less than 80 Å^2^. The final model includes 341 water molecules and two calcium ions. R_work_ and R_free_ converged to 17.1% and 20.6%, respectively. Model assessment and validation were carried out by PROCHECK^28^ and showed 99.7% of the residues in most favored and 0.3% in the allowed regions of the Ramachandran plot. The structure was deposited in the Protein Data Bank under the accession code: 4u3s. The crystal structure of the *Ac*CohScaB3-XDocScaAN178G complex from *A. cellulolyticus* was similarly phased by molecular replacement using BALBES^27^ with PDB ID code 2b59 from *C. thermocellum* as model. Data, up to 1.93 Å resolution, were used for structure refinement and the final statistics are summarized in Table S8. Initial building of the complex into the electron density was also performed using ARPwARP^23^ and manually built using COOT^24^. Refinement was done with REFMAC5^23^ and a final run of PDB_REDO. Water molecules were added/validated according to the same criteria. The final model contains 219 water molecules and two calcium ions. R_work_ and R_free_ converged to 19.7% and 24.1%, respectively. Model assessment and validation using the above-mentioned software revealed a model with 99.1% of the residues in most favored and 0.9% in the allowed regions of the Ramachandran plot. The corresponding set of coordinates is deposited in the Protein Data Bank under the accession code 4wi0. In both complexes, the molecules in the asymmetric unit are arranged as a heterodimer composed of chains A/B. In both structures all atoms in the cohesin (chain A) could be properly assigned and refined, apart from the first residue of chain A of *Ac*CohScaB3-XDocScaAN145G (the initiating engineered methionine). While on the XDoc (chain B) the exceptions were the first 28 residues in both complexes (23 residues coming from the cloning and expression tag constructs and the following initial 5 residues from the X-module). Data collection and refinement details data for the two complete structures are summarised in Table S8.

### Data availability

Structures described in this manuscript have been deposited in Protein Data Bank under accession codes 5m0y, 5k39, 5g5d, 4u3s, 4wi0. The authors declare that all other data supporting the findings of this study are included in the manuscript and its Supplementary Files or are available from the corresponding authors upon request.

## Additional Information

**How to cite this article**: Brás, J. L. A. *et al*. Diverse specificity of cellulosome attachment to the bacterial cell surface. *Sci. Rep.* **6**, 38292; doi: 10.1038/srep38292 (2016).

**Publisher's note:** Springer Nature remains neutral with regard to jurisdictional claims in published maps and institutional affiliations.

## Supplementary Material

Supplementary Information

Supplementary Tables

## Figures and Tables

**Table 1:** Thermodynamics of the interaction of CipB XDoc and its mutant derivatives with Cohs of ScaE6 and ScaC2.

Cohesin	Dockerin	*K*_*a*_ *M*^−1^	*ΔG kcal mol*^*−1*^	*ΔH kcal mol*^*−1*^	*TΔS kcal mol*^*−1*^	*N*
ScaE6	CipB XDoc	1.21E7 ± 2.96E6	−6.23 ± 0.19	−8.27 ± 0.19	−2.04	1.00 ± 0.03
	M114A	7.17E6 ± 6.25E5	−9.99 ± 1.06	−12.99 ± 1.06	−3.00	1.01 ± 0.04
	M118A	6.96E6 ± 1.73E5	−9.95 ± 0.62	−25.54 ± 0.62	−15.59	0.99 ± 0.05
	S121A	9.16E6 ± 2.49E5	−3.72 ± 0.16	−6.93 ± 0.16	−3.21	1.01 ± 0.06
	F124A	*Nd*	*Nd*	*Nd*	*Nd*	*Id*
	L147A	*Nd*	*Nd*	*Nd*	*Nd*	*Id*
	F148A	*Nd*	*Nd*	*Nd*	*Nd*	*Id*
	I154A	1.08E7 ± 1.92E6	−10.25 ± 0.94	−17.26 ± 0.94	−7.01	1.03 ± 0.08
ScaC2	CipB XDoc	*Id*	−11.32 ± 0.12	−12.39 ± 0.12	1.07	*Id*
	M114A	*Id*	−11.83 ± 0.39	−10.43 ± 0.39	1.40	*Id*
	M118A	*Id*	−11.57 ± 0.17	−8.66 ± 0.17	2.91	*Id*
	S120A	*Id*	−10.84 ± 0.28	−10.28 ± 0.28	0.56	*Id*
	F124A	*Nd*	*Nd*	*Nd*	*Nd*	*Id*
	L147A	*Nd*	*Nd*	*Nd*	*Nd*	*Id*
	F148A	6.19E5 ± 1.90E5	−8.42 ± 0.48	−8.83 ± 0.48	−0.42	1.01 ± 0.06
	I154A	1.20E6 ± 2.59E5	−9.13 ± 0.74	−9.75 ± 0.74	−0.63	1.02 ± 0.09

Interaction of CipB XDoc and all its seven mutant derivatives for ScaF Coh were too high to be quantified.

*Nd* means that the values were too low to be determined. *Id* impossible to determine.
