# Supplementary material for: Diverse specificity of cellulosome attachment to the bacterial cell surface
Source: Sci Rep. 2016 Dec 7;6:38292. doi: 10.1038/srep38292 (PMC5141474; doi:10.1038/srep38292)
Supplement: Supplementary Information [file srep38292-s1.docx]

**Supplementary figures and tables**

**Diverse specificity of cellulosome attachment to the bacterial cell surface**

Joana L.A. Brás^1,2,a^, Benedita Pinheiro^1,3,a^, Kate Cameron^1,a^, Fiona Cuskin^4,a^, Aldino Viegas^3,5^, Shabir Najmudin^1^, Pedro Bule^1^, Virginia M.R. Pires^1^, Maria João Romão^3^, Edward A. Bayer^6^, Holly L. Spencer^7^, Steven Smith^7^, Harry J. Gilbert^4^, Victor D. Alves^1,*^, Ana Luísa Carvalho^3,*^ and Carlos M.G.A. Fontes^1,2,*^

^1^Centro Interdisciplinar de Investigação em Sanidade Animal, Faculdade de Medicina Veterinária, Universidade de Lisboa, 1300-477 Lisboa, Portugal; ^2^NZYTech Genes & Enzymes, Campus do Lumiar, Estrada do Paço do Lumiar, Edifício E, r/c, 1649-038 Lisboa, Portugal; ^3^UCIBIO-REQUIMTE, Departamento de Química, Faculdade de Ciências e Tecnologia, Universidade Nova de Lisboa, 2829-516 Caparica, Portugal; ^4^Institute for Cell and Molecular Biosciences, Newcastle University, The Medical School, Newcastle upon Tyne NE2 4HH, United Kingdom; ^5^Institute of Physical Biology, Heinrich Heine University, Universitätsstr. 1, 40225 Düsseldorf, Germany; ^6^Department of Biomolecular Sciences, The Weizmann Institute of Science, Rehovot, Israel; ^7^Department of Biomedical and Molecular Sciences, Queen's University, Kingston, ON K7L 3N6, Canada.


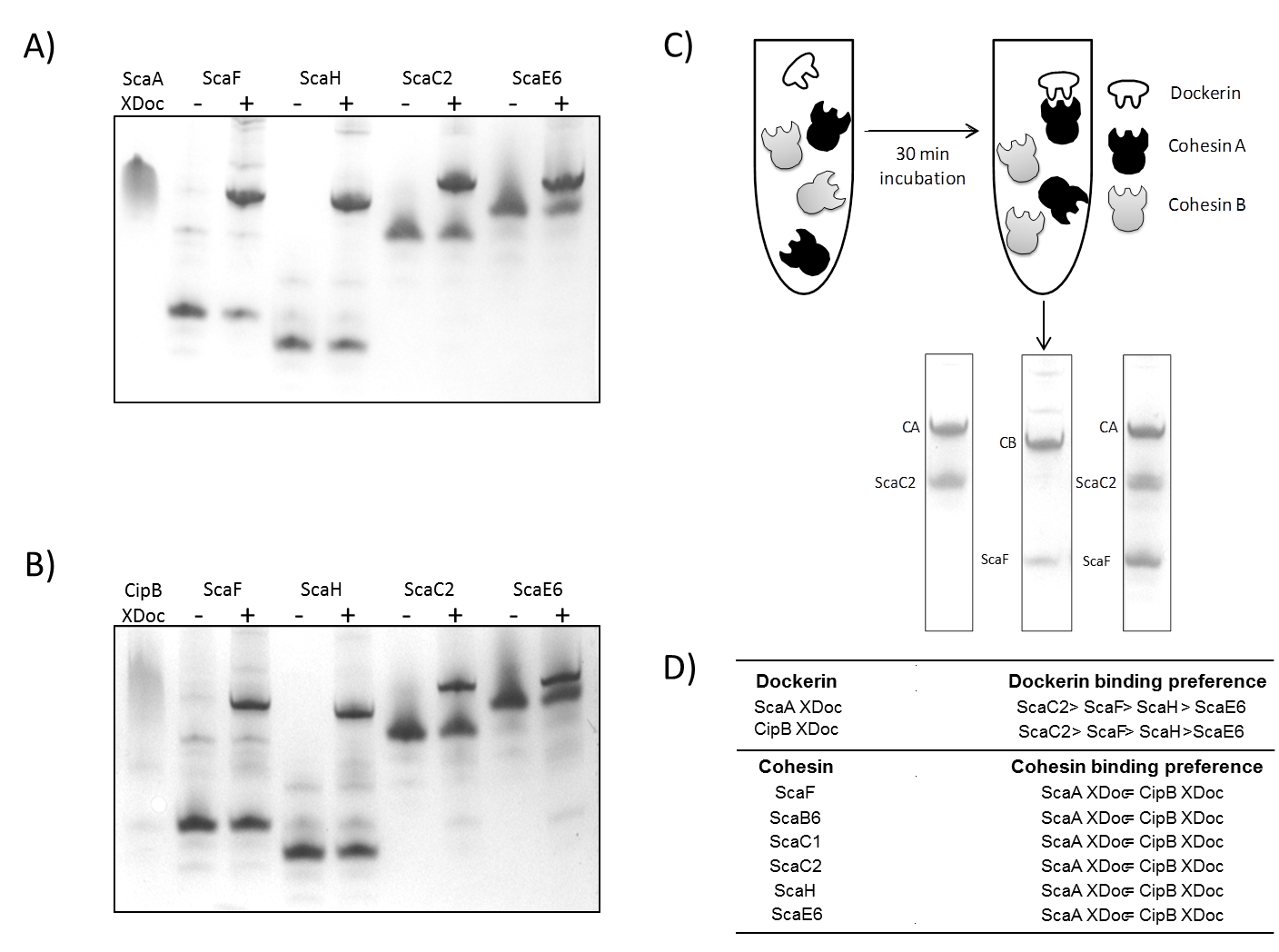


Figure S1 - Detection of *C. thermocellum* type II Coh-Doc specificities and binding preferences as evaluated by non-denaturing gel electrophoresis. A) XDoc module of ScaA was probed against the Cohs of ScaF, ScaC, ScaH and ScaE. B) XDoc module of CipB was probed against the Cohs of ScaF, ScaC, ScaH and ScaE. In panels A and B cohesins were used at a double molar concentration in relation to dockerins. C) The method used to detect preferential partners for dockerin is illustrated. The Doc is mixed with a double molar concentration of two potential Coh partners, and after a 30-min incubation period the complex formed is visualized through non-denaturing gel electrophoresis. An example of one such experiment is shown, where ScaA XDoc is mixed with ScaC2 Coh, forming complex A (CA) or ScaF Coh, forming complex B (CB). When the XDoc module is mixed with the two Cohs exclusively, complex A (CA) is formed, revealing that the ScaA XDoc module displays a preference for binding to ScaC2. D) The method described in panel C) was used to identify preferred Coh and Doc partners as listed.

Figure S2 - *Ct*CohScaC2-XDocCipB and *Ct*CohScaF-XDocScaA complex interfaces between the dockerin and the X module (A and B) and between the dockerin and the cohesin (C, D, E and F). A), C) and E) correspond to the *Ct*CohScaC2-XDocCipB complex. B), D) and F) correspond to the *Ct*CohScaF-XDocScaA complex.


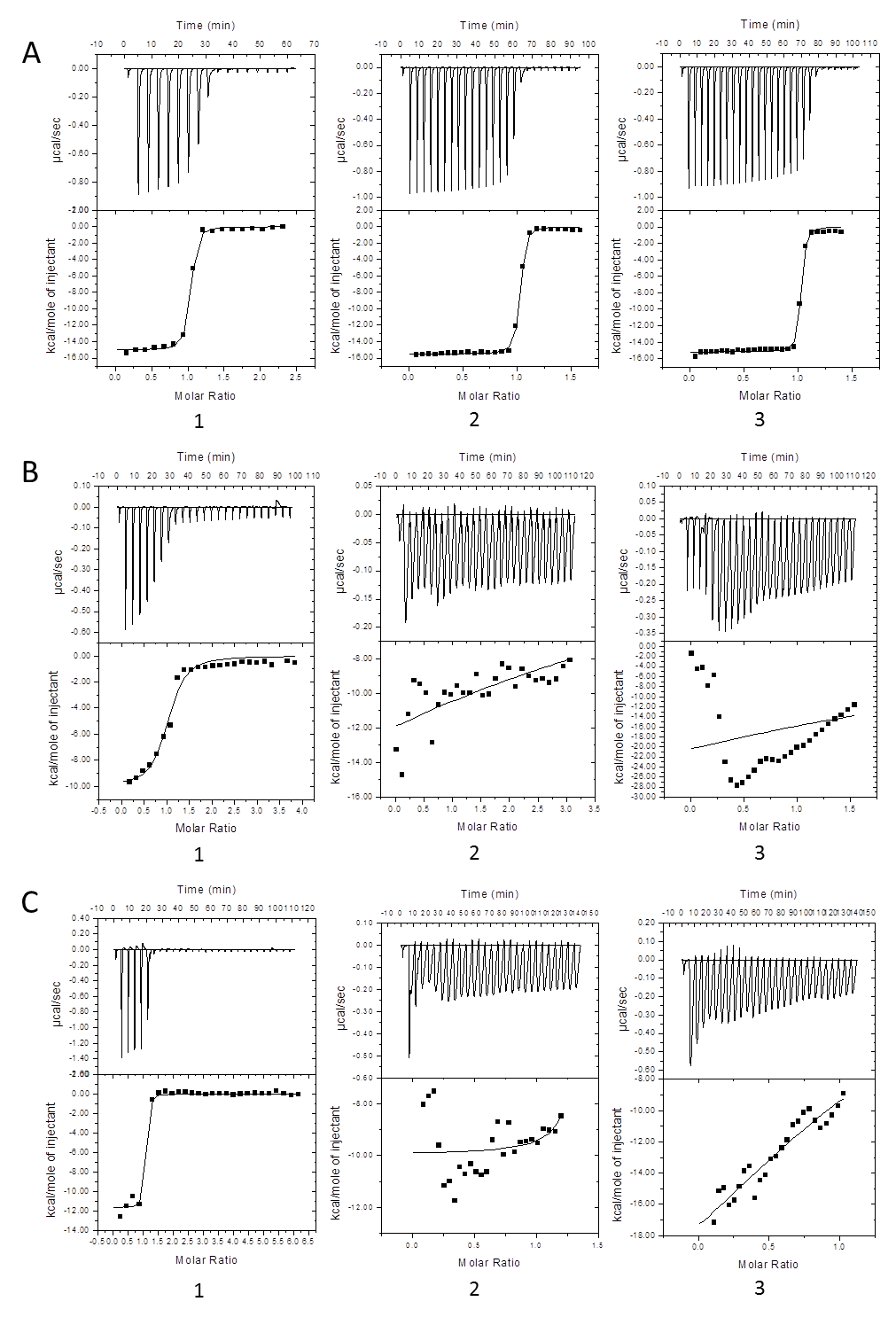


Figure S3 - Examples of the isothermal titration calorimetry (ITC) experiments performed using wild-type CipB Xdoc, its mutant derivatives Phe124A and Leu147A and the wild-type cohesins ScaF A), ScaE6 B) and ScaC2 C). The upper parts of each panel show the raw heats of binding, whereas the lower parts are the integrated heats after correction for heat of dilution. The curve represents the best fit to a single-site binding model. 1) Coh plus wild-type CipB XDoc. 2) Coh plus CipB XDoc Phe124A. 3) Coh plus CipB XDoc Leu147A.


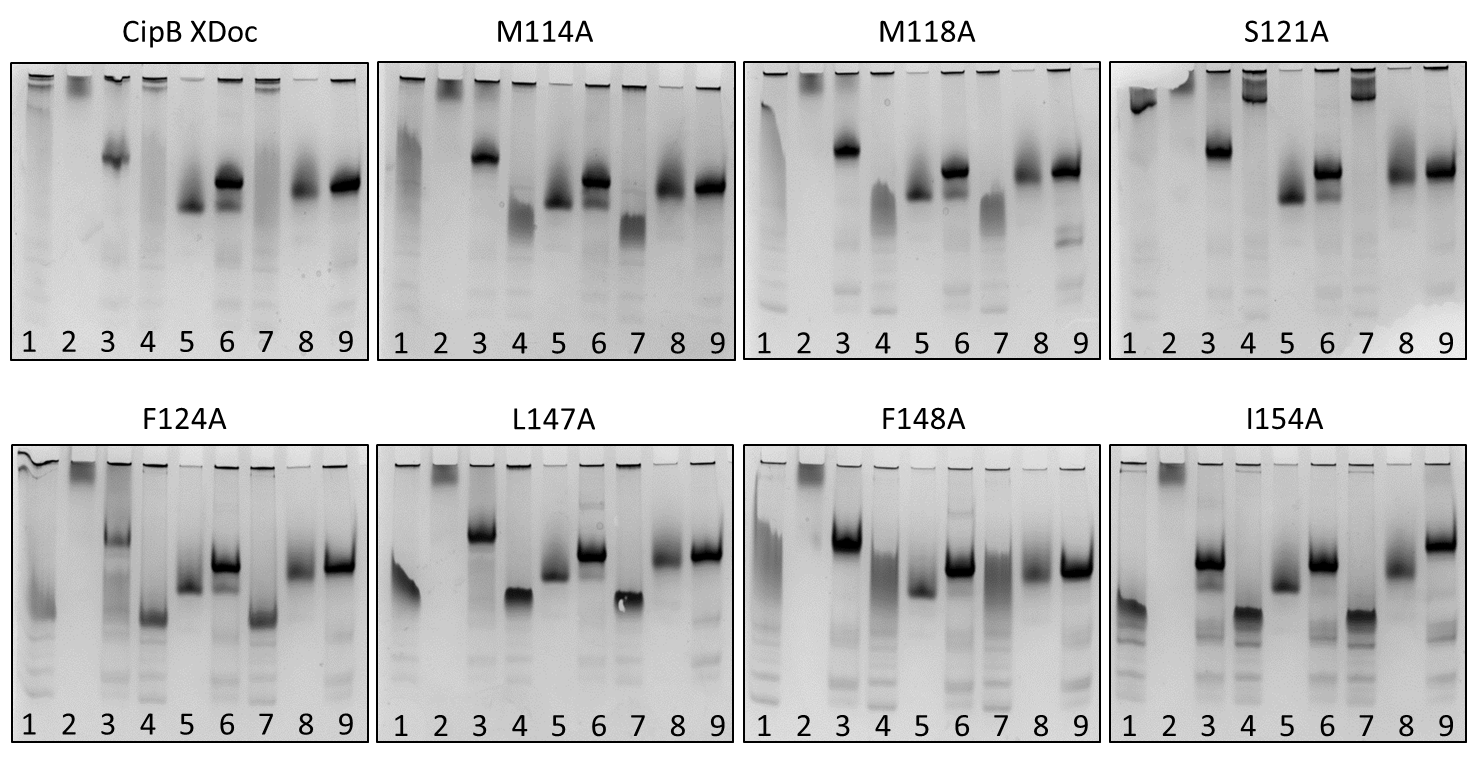


Figure S4 – Interaction of CipB XDoc and its seven mutant derivatives with ScaF, ScaC2 and ScaE6 at 45 ºC with complex formation probed by non-denaturing gel electrophoresis. The dockerins were loaded in lanes 1, 4 and 7. The cohesins were loaded in lanes 2 (ScaE6), 5 (ScaF) and 8 (ScaC2). Complexes were loaded in lanes 3 (Doc with ScaE6), 6 (Doc with ScaF) and 9 (Doc with ScaC2).

**Table S3** – Primary sequences of cohesins and dockerins involved in the formation of protein complexes

| Protein complex | Cohesin | Dockerin | PDB |
| --- | --- | --- | --- |
| *Ct*CohScaC2-XDocCipB | MASAHIALELDKTKVKVGDVIVATVKAKNMTSMAGIQVNIKYDPEVLQAIDPATGKPFTKETLLVDPELLSNREYNPLLTAVNDINSGIINYASCYVYWDSYRESGVSESTGIIGKVGFKVLKAANTTVKLEETRFTPNSIDGTLVIDWYGQQIVGYKVIQPDLEHHHHHH | MNNDSTDKTTVSGYISVDFDYPPESESKIKSGFNVKVAGTELSTKTDEKGYFEISGIPGDMREFTLEISKRNYLKRNVTVNGTGKLVVSTEDNPLILWAGDVERKGVQDNAINMVDVMEISKVFGTRAGDEEYVAELDLNMDGAINLFDIAIVIRHFNALPSRY | 5k39 |
| *Ct*CohScaC2-XDocScaA | MASAHIALELDKTKVKVGDVIVATVKAKNMTSMAGIQVNIKYDPEVLQAIDPATGKPFTKETLLVDPELLSNREYNPLLTAVNDINSGIINYASCYVYWDSYRESGVSESTGIIGKVGFKVLKAANTTVKLEETRFTPNSIDGTLVIDWYGQQIVGYKVIQPDLEHHHHHH | MNKPVIEGYKVSGYILPDFSFDATVAPLVKAGFKVEIVGTELYAVTDANGYFEITGVPANASGYTLKISRATYLDRVIANVVVTGDTSVSTSQAPIMMWVGDIVKDNSINLLDVAEVIRCFNATKGSANYVEELDINRNGAINMQDIMIVHKHFGATSSDYDAQ | 5g5d |
| *Ct*CohScaF-XDocCipB | MASRADKASSIELKFDRNKGEVGDILIGTVRINNIKNFAGFQVNIVYDPKVLMAVDPETGKEFTSSTFPPGRTVLKNNAYGPIQIADNDPEKGILNFALAYSYIAGYKETGVAEESGIIAKIGFKILQKKSTAVKFQDTLSMPGAISGTQLFDWDGEVITGYEVIQPDVLSLGDEPYEVEHHHHHH | MNNDSTDKTTVSGYISVDFDYPPESESKIKSGFNVKVAGTELSTKTDEKGYFEISGIPGDMREFTLEISKRNYLKRNVTVNGTGKLVVSTEDNPLILWAGDVERKGVQDNAINMVDVMEISKVFGTRAGDEEYVAELDLNMDGAINLFDIAIVIRHFNALPSRY | 5g5b |
| *Ac*CohScaB3-XDocScaA | MESYITMNFDKNTAEVGQIIKATVKINKITNFSGYQVNIKYDPTVLQAVNPKTGVAYTNSSLPTSGELLVNEDYGPIVQGVHKISEGILNLSRSYTALDVYRASESPEETGTVAVVGFKALQKKATTVVFEHSVTMPNGIIGTTLFNWYGNRITSGYSVIQPGEINSE | MGSSHHHHHHSSGLVPRGSHMASGIVSEGTTVSGYINPDFVTTSTTAPIVKAGFTVEIVGTTKSAVTDSNGYFEIKDVAAGTYTVKITKANYLTREIANVSVTADKELSTSASPILMWAGDMAIGGTQDGAINLEDILEICKAFNTSSTDAKYQVGLDLNRDGAISLEDVMIVAKHFNKVSSDY | Nd |
| *Ac*CohScaB3-XDocScaAN145G | MESYITMNFDKNTAEVGQIIKATVKINKITNFSGYQVNIKYDPTVLQAVNPKTGVAYTNSSLPTSGELLVNEDYGPIVQGVHKISEGILNLSRSYTALDVYRASESPEETGTVAVVGFKALQKKATTVVFEHSVTMPNGIIGTTLFNWYGNRITSGYSVIQPGEINSE | MGSSHHHHHHSSGLVPRGSHMASGIVSEGTTVSGYINPDFVTTSTTAPIVKAGFTVEIVGTTKSAVTDSNGYFEIKDVAAGTYTVKITKANYLTREIANVSVTADKELSTSASPILMWAGDMAIGGTQDGAINLEDILEICKAFGTSSTDAKYQVGLDLNRDGAISLEDVMIVAKHFNKVSSDY | 4u3s |
| *Ac*CohScaB3-XDocScaAN178G | MESYITMNFDKNTAEVGQIIKATVKINKITNFSGYQVNIKYDPTVLQAVNPKTGVAYTNSSLPTSGELLVNEDYGPIVQGVHKISEGILNLSRSYTALDVYRASESPEETGTVAVVGFKALQKKATTVVFEHSVTMPNGIIGTTLFNWYGNRITSGYSVIQPGEINSE | MGSSHHHHHHSSGLVPRGSHMASGIVSEGTTVSGYINPDFVTTSTTAPIVKAGFTVEIVGTTKSAVTDSNGYFEIKDVAAGTYTVKITKANYLTREIANVSVTADKELSTSASPILMWAGDMAIGGTQDGAINLEDILEICKAFNTSSTDAKYQVGLDLNRDGAISLEDVMIVAKHFGKVSSDY | 4wi0 |

Nd, not determined

**Table S5** – Primary sequence of recombinant cohesins and dockerins produced in the present study. Genes encoding cohesins were cloned into pET28 (NheI-XhoI), and the corresponding recombinant protein contains a N-terminal His6 tag. XDockerin genes (encoding an X module fused to the dockerin) were cloned into pET21a (NdeI -XhoI), and the recombinant protein contains an engineered C-terminal His6 tag.

| Protein | Name | Organism | Primary Sequence |
| --- | --- | --- | --- |
| Cohesin | ScaF | *Clostridium thermocellum* | DKASSIELKFDRNKGEVGDILIGTVRINNIKNFAGFQVNIVYDPKVLMAVDPETGKEFTSSTFPPGRTVLKNNAYGPIQIADNDPEKGILNFALAYSYIAGYKETGVAEESGIIAKIGFKILQKKSTAVKFQDTLSMPGAISGTQLFDWDGEVITGYEVIQPD |
|  | ScaB6 | *Clostridium thermocellum* | DSYVIMELDKTKVKVGDIITATIKIENMKNFAGYQLNIKYDPTMLEAIELETGSAIAKRTWPVTGGTVLQSDNYGKTTAVANDVGAGIINFAEAYSNLTKYRETGVAEETGIIGKIGFRVLKAGSTAIRFEDTTAMPGAIEGTYMFDWYGENIKGYSVVQPG |
|  | ScaC1 | *Clostridium thermocellum* | SRISMELDKTKANIGDIIIATIRIDNINNFSGYQLNIKYDPSYLQAVNPLTGEPIKKRTMPAVNGTVLLKGDQYSITEVVENNVDEGILNFGKGYANLTEYRKSGKPETTGIIGKIGFKALKLGKTEIKFENTPVMPGAKEGTLLFDWDAETITEYNVIQP |
|  | ScaC2 | *Clostridium thermocellum* | AHIALELDKTKVKVGDVIVATVKAKNMTSMAGIQVNIKYDPEVLQAIDPATGKPFTKETLLVDPELLSNREYNPLLTAVNDINSGIINYASCYVYWDSYRESGVSESTGIIGKVGFKVLKAANTTVKLEETRFTPNSIDGTLVIDWYGQQIVGYKVIQPD |
|  | ScaH | *Clostridium thermocellum* | AEANIQIVLDKNTAKKDEIITAKIILNNIPKIAGYQVNIKYDPNILQAVDLDTGKPLEDKQIPGGGDVLSNPDYNVLPLAASDVKNGVINFAKAYVNVDEYKESNNPESSGVLALIGFKVLKEESTVISFADTPSMPNAVSGTYVYDWDFNVLTNYSVGKGVKVN |
|  | ScaE6 | *Clostridium thermocellum* | YIKLEFDKNTASEGEIIRATVKVNNVKNLAGYQICIKYDPNVLQPVNPNTGAAYTTTTHLVDGELIVKQEYGSTSMAAHRLSNGILNFARTYLYVSDYKEDGKPEETGILGVIGFKVLKKEKTTVSFYADEALMPNSVSGTYLIDWNSNKKTDYKVIQP |
|  | ScaB3 | *Acetivibrio cellulolyticus* | ESYITMNFDKNTAEVGQIIKATVKINKITNFSGYQVNIKYDPTVLQAVNPKTGVAYTNSSLPTSGELLVNEDYGPIVQGVHKISEGILNLSRSYTALDVYRASESPEETGTVAVVGFKALQKKATTVVFEHSVTMPNGIIGTTLFNWYGNRITSGYSVIQPGEINSE |
| XDockerin | ScaA | *Clostridium thermocellum* | MNKPVIEGYKVSGYILPDFSFDATVAPLVKAGFKVEIVGTELYAVTDANGYFEITGVPANASGYTLKISRATYLDRVIANVVVTGDTSVSTSQAPIMMWVGDIVKDNSINLLDVAEVIRCFNATKGSANYVEELDINRNGAINMQDIMIVHKHFGATSSDYDAQ |
|  | CipB | *Clostridium thermocellum* | MNNDSTDKTTVSGYISVDFDYPPESESKIKSGFNVKVAGTELSTKTDEKGYFEISGIPGDMREFTLEISKRNYLKRNVTVNGTGKLVVSTEDNPLILWAGDVERKGVQDNAINMVDVMEISKVFGTRAGDEEYVAELDLNMDGAINLFDIAIVIRHFNALPSRY |
|  | ScaA | *Acetivibrio cellulolyticus* | GIVSEGTTVSGYINPDFVTTSTTAPIVKAGFTVEIVGTTKSAVTDSNGYFEIKDVAAGTYTVKITKANYLTREIANVSVTADKELSTSASPILMWAGDMAIGGTQDGAINLEDILEICKAFNTSSTDAKYQVGLDLNRDGAISLEDVMIVAKHFNKVSSDY |

**Table S6** – Primers used to produce dockerin mutant derivatives obtained in the present study.

| **Dockerin derivative** | **Sequence (5’ 🡪 3’)** | **Direction** |
| --- | --- | --- |
| XDocCipB M114A | GCAAGACAATGCTATTAAT**GC**GGTGGATGTGATGGAAATATCC | Forward |
|  | GGATATTTCCATCACATCCACC**GC**ATTAATAGCATTGTCTTGC | Reverse |
| XDocCipB M118A | GCTATTAATATGGTGGATGTG**GC**GGAAATATCCAAAG | Forward |
|  | CTTTGGATATTTCC**GC**CACATCCACCATATTAATAGC | Reverse |
| XDocCipB S121A | GTGGATGTGATGGAAATA**G**CCAAAGTTTTTGGCAC | Forward |
|  | GTGCCAAAAACTTT**GGC**TATTTCCATCACATCCAC | Reverse |
| XDocCipB F124A | GGAAATATCCAAAGTT**GC**TGGCACAAGAGCCGGAGATG | Forward |
|  | CATCTCCGGCTCTTGTGCCA**GC**AACTTTGGATATTTCC | Reverse |
| XDocCipB L147A | GGACGGAGCAATCAAT**GC**ATTTGATATAGCTATAGTTATCAGGC | Forward |
|  | GCCTGATAACTATAGCTATATCAAAT**GC**ATTGATTGCTCCGTCC | Reverse |
| XDocCipB F148A | GGACGGAGCAATCAATTTA**GC**TGATATAGCTATAGTTATCAGGC | Forward |
|  | GCCTGATAACTATAGCTATATCA**GC**TAAATTGATTGCTCCGTCC | Reverse |
| XDocCipB I154A | GATATAGCTATAGTT**GA**CAGGCATTTTAACGCATTACC | Forward |
|  | GGTAATGCGTTAAAATGCCTG**TC**AACTATAGCTATATC | Reverse |

**Table S7** – Data collection and refinement statistics of *C. thermocellum* coh-doc complexes.

| ***Coh-XDoc complex*** | | ***CtCohScaF-XDocCipB*** | ***CtCohScaC2-XDocCipB*** | ***CtCohScaC2-XDocScaA*** |
| --- | --- | --- | --- | --- |
| Space Group | | P2_1_2_1_2_1_ | C121 | I222 |
| Unit cell parameters  a, b, c (Å) α, β, γ (°) | | 43.4, 63.7, 141.2  90.0, 90.0, 90.0 | 116.7, 78.6, 35.8  90.0, 95.8, 90.0 | 52.4, 125.8, 130.5  90.0, 90.0, 90.0 |
| Matthews parameter (Å^3^/Da) | | 2.6 | 2.2 | 3.1 |
| ***Data collection statistics*** | |  |  |  |
| X-ray source | | ESRF, ID29 | ESRF, ID14-4 | ESRF, ID29 |
| Wavelength (Å) | | 0.9762 | 0.9735 | 0.9537 |
| No. of unique reflections | | 63360 | 21909 | 8989 |
| Resolution limits (Å) | | 47.31 – 1.5 (1.53-1.50) | 39.31 – 1.98 (2.09 – 1.98) | 45.28 – 3.00 (3.18 – 3.00) |
| Completeness (%) | | 99.5 (99.6) | 97.8 (98.2) | 100 (100) |
| Redundancy | | 4.4 (4.5) | 3.6 (3.7) | 7.0 (7.2) |
| Average I/σ(I) | | 18.8 (2.3) | 13.10 (5.5) | 5.9 (1.5) |
| R_merge_ (%) | | 3.7 (64.8) | 9.4 (17.3) | 18.9 (118) |
| R_pim_ (%) | | 2.0 (33.8) | 5.8 (10.4) | 8.2 (50.1) |
| Half-dataset correlation CC(1/2) | | 0.999 (0.736) | not determined | 0.991 (0.737) |
| ***Refinement statistics*** | |  |  |  |
| Resolution limits (Å) | | 47.3 - 1.5 | 18.8 – 1.98 | 48.3 – 3.0 |
| R-work | | 0.190 | 0.187 | 0.255 |
| R-free | | 0.203 | 0.247 | 0.303 |
| No. protein residues in the asymmetric unit | | 333 | 320 | 316 |
| No. water molecules in the asymmetric unit | | 201 | 322 | 0 |
| No. atoms in the asymmetric unit | | 2855 | 2819 | 2420 |
| rmsd bond length (Å) | | 0.006 | 0.009 | 0.007 |
| rmsd bond angles (°) | | 1.243 | 1.154 | 1.20 |
| *Average temperature factor (Å^2^)* | |  |  |  |
| main chain | | 22.2 | 17.8 | 48.0 |
| side chain | | 24.2 | 18.7 | 48.4 |
| Calcium 1 | | 14.8 | 17.0 | 64.4 |
| Calcium 2 | | 19.5 | 14.6 | 62.3 |
| solvent molecules | | 34.9 | 36.8 | - |
| Ramachandran plot | outliers (%) | 0 | 0 | 0 |
|  | favored (%) | 98.5 | 97.0 | 95 |
| PDB ID codes | | 5m0y | 5k39 | 5g5d |

R_merge_ = [Σ |I-<I>|]/Σ <I>, where I is the observed intensity, and <I> is the statistically weighted average intensity of multiple observations.

R_pim_ = [Σ √(1/(n-1)) Σ |I-<I>|]/Σ <I>, a redundancy-independent version of R_merge_

R_work_ = Σ ||F_calc_|− |F_obs_||/Σ |F_obs_|× 100, where F_calc_ and F_obs_ are the calculated and observed structure factor amplitudes, respectively (R_free_ is calculated for a randomly chosen 5% of the reflections).

Geometry values from Molprobity.

Values in parentheses are for the highest resolution shell.

**Table S8** – Data collection and refinement statistics of *A. cellulolyticus* coh-doc complexes.

| ***Coh-XDoc complex*** | | ***AcCohScaB3-XDocScaA_N145G*** | ***AcCohScaB3-XDocScaA_N178G*** |
| --- | --- | --- | --- |
| Space Group | | P6_5_22 | P2_1_2_1_2_1_ |
| Unit cell parameters  a, b, c (Å) α, β, γ (°) | | 72.3, 72.3, 231.5 90.0, 90.0, 120.0 | 38.5, 88.3, 92.0 90.0, 90.0, 90.0 |
| Matthews parameter (Å^3^/Da) | | 2.3 | 2.3 |
| ***Data collection statistics*** | |  |  |
| X-ray source | | Diamond, IO3 | ESRF, ID29 |
| Wavelength (Å) | | 0.976 | 0.976 |
| No. of unique reflections | | 45113 | 29281 |
| Resolution limits (Å) | | 62.63 – 1.64 (1.68 – 1.64) | 92.02 – 1.93 (1.98 – 1.93) |
| Completeness (%) | | 100 (100) | 90.8 (92.3) |
| Redundancy | | 26.7 (29.5) | 3.3 (3.1) |
| Average I/σ(I) | | 13.5 (3.2) | 5.0 (2.0) |
| R_merge_ (%) | | 11.3 (70.2) | 13.6 (49.7) |
| R_pim_ (%) | | 2.7 (13.0) | 8.0 (30.4) |
| Half-dataset correlation CC(1/2) | | 0.998 (0.856) | 0.987 (0.561) |
| ***Refinement statistics*** | |  |  |
| Resolution limits (Å) | | 62.6 – 1.64 | 63.7 – 1.93 |
| R-work | | 0.171 | 0.197 |
| R-free | | 0.206 | 0.241 |
| No. protein residues in the asymmetric unit | | 323 | 324 |
| No. water molecules in the asymmetric unit | | 341 | 219 |
| No. atoms in the asymmetric unit | | 2843 | 2680 |
| rmsd bond length (Å) | | 0.009 | 0.006 |
| rmsd bond angles (°) | | 1.371 | 1.097 |
| Average temperature factor (Å^2^) | |  |  |
| main chain | | 25.5 | 23.3 |
| side chain | | 28.3 | 24.3 |
| Calcium 1 | | 16.2 | 19.6 |
| Calcium 2 | | 19.7 | 19.9 |
| solvent molecules | | 31.9 | 28.9 |
| Ramachandran plot | outliers (%) | 0 | 0 |
|  | favored (%) | 98.5 | 98.5 |
| PDB ID codes | | 4u3s | 4wi0 |

R_merge_ = [Σ |I-<I>|]/Σ <I>, where I is the observed intensity, and <I> is the statistically weighted average intensity of multiple observations.

R_pim_ = [Σ √(1/(n-1)) Σ |I-<I>|]/Σ <I>, a redundancy-independent version of R_merge_.

R_work_ = Σ ||F_calc_|− |F_obs_||/Σ |F_obs_|× 100, where F_calc_ and F_obs_ are the calculated and observed structure factor amplitudes, respectively (R_free_ is calculated for a randomly chosen 5% of the reflections).

Geometry values from Molprobity.

Values in parentheses are for the highest resolution shell.
